# Gene amplifications cause high-level resistance against albicidin in gram-negative bacteria

**DOI:** 10.1371/journal.pbio.3002186

**Published:** 2023-08-10

**Authors:** Mareike Saathoff, Simone Kosol, Torsten Semmler, Karsten Tedin, Nicole Dimos, Johannes Kupke, Maria Seidel, Fereshteh Ghazisaeedi, Micela Condor Jonske, Silver A. Wolf, Benno Kuropka, Wojciech Czyszczoń, Dmitry Ghilarov, Stefan Grätz, Jonathan G. Heddle, Bernhard Loll, Roderich D. Süssmuth, Marcus Fulde

**Affiliations:** 1 Institute of Microbiology and Epizootics, Freie Universität Berlin, Berlin, Germany; 2 Institut für Chemie, Technische Universität Berlin, Berlin, Germany; 3 Robert Koch-Institute (RKI), MF2—Genome Sequencing and Genomic Epidemiology, Berlin, Germany; 4 Institute for Chemistry and Biochemistry, Freie Universität Berlin, Berlin, Germany; 5 Malopolska Centre of Biotechnology, Jagiellonian University, Krakow, Poland; 6 Veterinary Centre for Resistance Research (TZR), Freie Universität Berlin, Berlin, Germany; Biological Research Centre, HUNGARY

## Abstract

Antibiotic resistance is a continuously increasing concern for public healthcare. Understanding resistance mechanisms and their emergence is crucial for the development of new antibiotics and their effective use. The peptide antibiotic albicidin is such a promising candidate that, as a gyrase poison, shows bactericidal activity against a wide range of gram-positive and gram-negative bacteria. Here, we report the discovery of a gene amplification–based mechanism that imparts an up to 1000-fold increase in resistance levels against albicidin. RNA sequencing and proteomics data show that this novel mechanism protects *Salmonella Typhimurium* and *Escherichia coli* by increasing the copy number of STM3175 (YgiV), a transcription regulator with a GyrI-like small molecule binding domain that traps albicidin with high affinity. X-ray crystallography and molecular docking reveal a new conserved motif in the binding groove of the GyrI-like domain that can interact with aromatic building blocks of albicidin. Phylogenetic studies suggest that this resistance mechanism is ubiquitous in gram-negative bacteria, and our experiments confirm that STM3175 homologs can confer resistance in pathogens such as *Vibrio vulnificus* and *Pseudomonas aeruginosa*.

## Abstract 2

Antibiotic resistance is a continuously increasing concern for public health care. This study shows that transient gene amplification identifies a conserved transcriptional regulator as a resistance determinant against the peptide antibiotic albicidin in Gram-negative bacteria.

## Introduction

Antimicrobial resistance (AMR) in pathogenic, commensal and food-borne bacteria remains a major health hazard for humans. According to a recent study, in 2019, almost 5 million human deaths were estimated to be associated with bacterial AMR, with a prognosis of an increase of up to 10 million deaths by 2050. It is widely accepted that inappropriate use of antimicrobials as therapeutics and feed additives in human and veterinary medicine coupled with a lack in understanding of bacterial resistance mechanisms account for the worldwide increase of resistant and multiresistant bacteria [1,2]. To develop effective long-term solutions to combat AMR and improve public health, understanding resistance mechanisms and their evolution is crucial.

Bacterial adaptation to stress conditions occurs via different types of responses. In addition to stable genetic, bona fide resistance mechanisms, transient and unstable responses conferred, e.g., by differential gene regulation and protein stability, and changes in gene copy numbers, so-called gene duplication–amplification (GDA) events, are frequently observed [3–5]. The latter occur rapidly (e.g., <10 generations), whereas establishing genetic modifications takes thousands of generations. GDAs have been observed as a consequence of selective pressure from antibiotic exposure, often resulting in elevated cellular concentrations of a modifying or degrading enzyme, or of efflux pumps. In addition to their role in adapting bacteria quickly to harmful conditions, GDAs have also been implicated in the evolution of bona fide resistance mechanisms on a populational [5] and on a subpopulational level (so-called heteroresistance) [6]. However, as knowledge about the occurrence of GDAs in antibiotic treatment is still scarce and their verification in routine diagnostics is difficult, it must be assumed that GDAs have a nonnegligible impact on the success of antibiotic treatment of infectious diseases.

The promising antibacterial peptide albicidin was first isolated as a phytotoxic compound from the plant-pathogenic bacterium *Xanthomonas albilineans* [7,8]. Since then, albicidin [9–11] as well as the related compounds cystobactamids [12,13] and coralmycins [14,15] are undergoing development toward clinical use. Albicidin is active at nanomolar concentrations against a range of different gram-positive and gram-negative bacterial species, including *Klebsiella aerogenes*, *Salmonella Typhimurium*, *Escherichia coli*, as well as the ESKAPE pathogens *Enterococcus faecium*, *Staphylococcus aureus*, *Klebsiella pneumonia*, *Acinetobacter baumanii*, *Pseudomonas aeruginosa*, and *Enterobacter* spp. The antibiotic peptide inhibits the supercoiling activity of bacterial DNA gyrase (topoisomerase II) and effectively traps the covalent complex formed between DNA and gyrase at concentrations below those of most coumarin antibiotics and quinolones [16,17]. Several albicidin resistance mechanisms have been described, e.g., the ABC transporter AlbF that confers autoresistance in *Xanthomonas* via the active efflux of albicidin [18]. Further strategies comprise albicidin degradation by the endopeptidase AlbD produced by *Pantoea dispersa* by specifically cleaving a benzamide bond [19] or albicidin binding through the MerR-like transcriptional regulator AlbA synthesized by *Klebsiella oxytoca* [20]. In *E*. *coli*, adaption to albicidin is frequently linked to mutations in the nucleoside-specific transporter Tsx, resulting in the loss of active transport of albicidin across the outer membrane [21,22].

Here, we report that exposure of *S*. *Typhimurium* and *E*. *coli* to increasing concentrations of the gyrase poison albicidin results in chromosomal duplication–amplification. The affected region harbors the GyrI-like domain containing transcription regulator STM3175 (YgiV in *E*. *coli*), which we identify as a critical factor for conferring high-level resistance against albicidin. We further illustrate that this resistance mechanism is ubiquitous in gram-negative bacterial species with STM3175 homologs conferring resistance in *Vibrio* and *Pseudomonas*.

## Results

### Exposure to increasing albicidin concentration leads to Tsx transporter mutations and results in gene duplications

**Fig 1:**
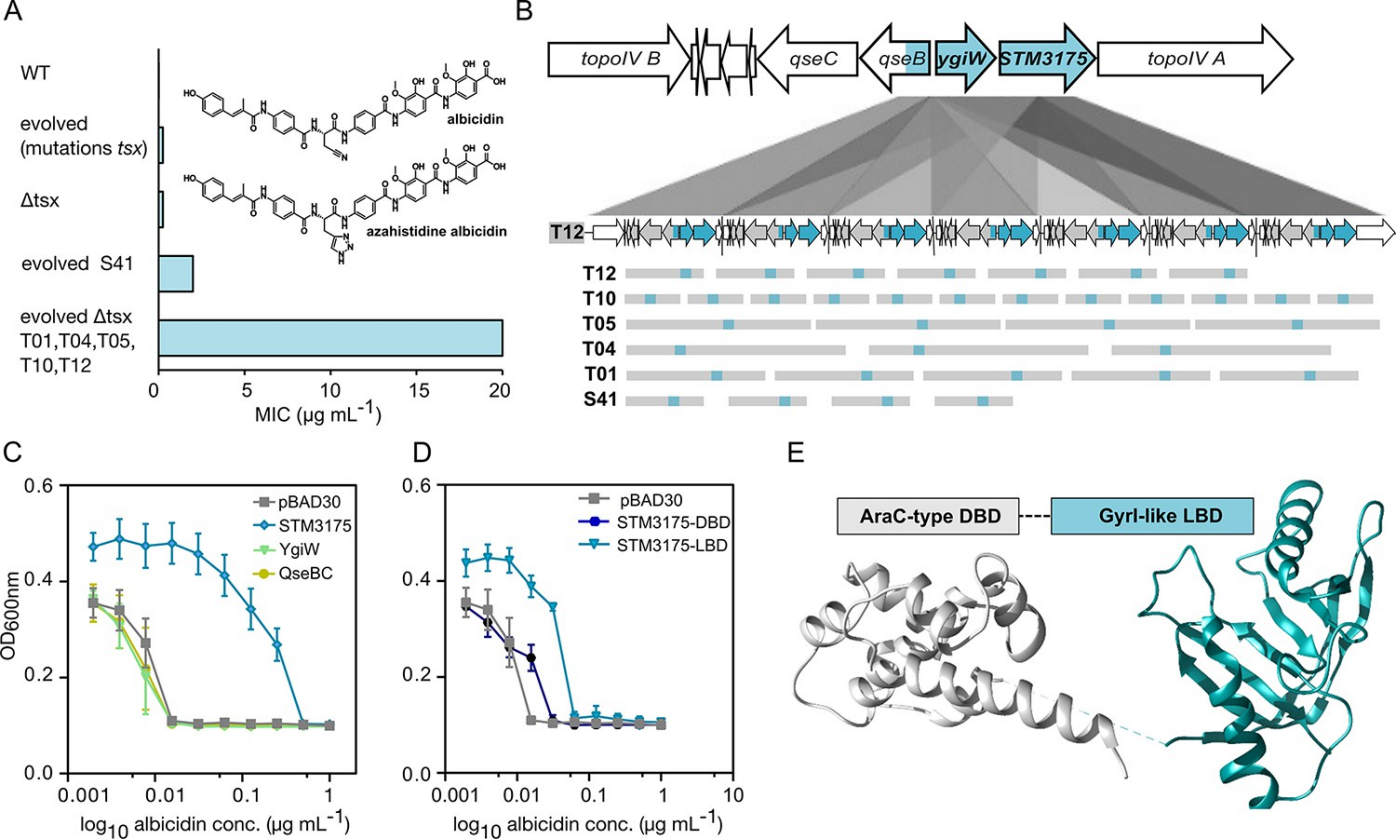
Albicidin high-level resistance resulting from GDAs in evolved *S*. *Typhimurium*. (**A**) Comparison of albicidin MICs for WT and evolved *S*. *Typhimurium* strains (see Fig C and Table B in S1 Text). A structure of albicidin and azahistidine albicidin is included in the bar diagram. (**B**) Nanopore sequencing revealed 7 copies of the GDA region in the evolved *tsx*-deficient *S*. *Typhimurium* strain T12 (see below, T12), including the genes between topoisomerase IV subunits A and B (see above, WT). The common approximately 2,200 bp long region present in all 6 evolved strains (S41, T01, T04, T05, T10, and T12) is highlighted in cyan. It includes 3 genes: *STM3175*, *ygiW*, and partly *qseB*. (**C**) Arabinose-induced overexpression of STM3175 (cyan), but not YgiW (light green) or QseBC (olive), results in an elevated albicidin MIC in *S*. *Typhimurium* WT. (**D**) Arabinose-induced overexpression of the LBD (cyan), but not of the DBD (blue) of STM3175 revealed and increased MIC against albicidin in *S*. *Typhimurium*. (**E**) Crystal structure of STM3175 with the conserved AraC-type DBD and GyrI-like LBD shown in gray and cyan, respectively (please also see Fig M in S1 Text). Source data are provided as a source data file (**S1 Data**). DBD, DNA-binding domain; GDA, gene duplication–amplification; MIC, minimum inhibitory concentration; LBD, ligand-binding domain; WT, wild-type.

To investigate the evolution of albicidin resistance in *S*. *Typhimurium*, we exposed the wild-type (WT) strain to albicidin concentrations considerably higher than the MIC (0.06 μg ml^−1^) and monitored growth over a 24-h period. Because of its higher stability, we used the azahistidine analog of albicidin (Fig 1A), which has similar activity compared to natural albicidin [9]. However, control experiments using natural albicidin showed comparable results (Fig A in S1 Text). Out of 90 clones tested, 14 showed growth under these conditions (Fig C in S1 Text). Genome sequencing of these albicidin resistant strains revealed that the majority (9 strains) carried mutations in the *tsx* gene. These mutations were associated with a 16-fold increased MIC (minimum inhibitory concentration), compared to the WT, similar to the isogenic *tsx*-deficient control strain (Fig 1A and Fig C and Table A in S1 Text). Remarkably, one of the remaining 5 resistant strains, with an intact *tsx* gene (strain S41), exhibited a more than 100-fold elevated MIC and was able to tolerate concentrations as high as 2 μg mL^−1^ albicidin (Fig 1A). Analysis of DNA sequencing data of strain S41 revealed a gene amplification resulting in 3 to 4 copies of an approximately 47-kb genomic region but no single nucleotide polymorphisms (SNPs) (Tables A and B in S1 Text).

The GDAs caught our interest and we decided to investigate this mechanism in more detail. We therefore repeated the evolution experiments with a mutant strain lacking the *tsx* gene (Δ*tsx*) in order to avoid resistance effects resulting from mutations in the nucleoside transporter gene. After exposing the Δ*tsx* strain stepwise to increasing albicidin concentrations from 0.125 μg ml^−1^ to 20 μg ml^−1^ (Fig 1A), similar genome alterations were observed as in the *tsx-*proficient WT strains that adapted to the antibiotic. Whole genome sequencing revealed that 5 out of 10 tested strains harbored GDAs ranging between 3 kb and 158 kb (strains T01, T04, T05, T10, and T12; Fig D(A) and Table B in S1 Text) with copy numbers varying between 3 and 15. In these strains, either no SNPs (2/5: T01 and T10) or SNPs in genes concerning the common segment of the GDA region (3/5: T04, T05, and T12—*qseB*, *stm3175*, or *topoisomerase IV subunit B* and additionally one hypothetical protein (1/5: T12)) were identified (Table C in S1 Text), suggesting that this particular GDA region is responsible for the significantly increased albicidin tolerance in *S*. *Typhimurium*: 80-fold compared to the input strain or more than 1,000-fold compared to the WT strain (Fig 1A). As mentioned above, a hallmark of GDAs is their reversibility in the absence of the appropriate selection pressure. To test the persistence of the GDAs, we incubated the evolved strains (T01, T04, T05, T10, T12, and S41) as biological triplicates for 24 h without albicidin over a period of 6 (T01, T04, T05, T10, T12, and S41) and 15 days (T12) (equivalent to approximately 600 and 1,500 generations, respectively). As shown in Fig B and Table D in S1 Text, we observed a reduction in gene duplications in most of the cases, which correspondingly resulted in a lower MIC to albicidin (Table D in S1 Text). As the phenomenon of heteroresistance is a complex mechanism that happens at the single-cell level, and where compensation and biologic fitness play a role, further experiments are needed to elucidate in detail the genesis of albicidin-mediated GDAs.

### GDAs contain genes for the sensory histidine kinase system QseBC and the regulatory protein STM3175

Alignments of the GDAs revealed a conserved approximately 2,200 bp overlap between the amplified regions present in all 6 (S41, T01, T04, T05, T10, and T12) analyzed strains (Fig D in S1 Text). The overlapping region contains the 2 genes *STM3175* and *ygiW* that are transcribed polycistronically in an operon structure and the N-terminal part of *qseB* (Fig 1B and Fig D(B) in S1 Text). Interestingly, *STM3175* and *ygiW* are located directly upstream of the gene encoding DNA topoisomerase IV subunit A (Fig 1B and Fig D(B) in S1 Text). YgiW, a putative periplasmic protein has been linked to the oxidative stress response in *E*. *coli* [23]. Little is known about the function of STM3175, a putative regulatory protein. QseB and the adjacently encoded QseC (Fig 1B) belong to a 2-component quorum sensing system consisting of a sensory histidine kinase (QseC) and its response regulator (QseB). To investigate if the absence of these genes affects albicidin tolerance of *S*. *Typhimurium*, we constructed knockout mutants of *qseBC* and *STM3175-ygiW* of the WT and Δ*tsx* strain. In MIC assays and corresponding colony-forming unit (CFU) counts, no detectable significant change in albicidin sensitivity was observed compared to the parent strains (Fig E in S1 Text), suggesting that these genes only affected albicidin tolerance in GDAs and higher copy numbers.

### GDAs of the regulator STM3175 afford albicidin resistance

To investigate which of the amplified genes was responsible for the increased albicidin tolerance, MIC assays and corresponding CFU counts with *STM3175*, *ygiW*, and *qseB* were conducted under control of an arabinose-inducible promoter. Each gene was cloned into the low-copy number plasmid pBAD30 and expressed in *S*. *Typhimurium* WT cells. After induction with arabinose, only overexpression of STM3175 imparted resistance, whereas overexpression of YgiW and QseB did not elicit albicidin tolerance (Fig 1C and Fig F(A-B) and Fig F(E) in S1 Text). Notably, the level of albicidin resistance increased with increasing arabinose concentration (Fig G in S1 Text). This suggested that the resistance mechanism solely depended on the increase of STM3175 concentration, which would be elevated by the increased copy number of STM3175 in the GDAs. Indeed, RNA-Seq analysis showed a clear increase in mRNA levels of the *STM3175*-*ygiW* operon as well as the other constituents of the GDA, including *qseB/C* but not the flanking topoisomerase IV subunits A and B (Fig H in S1 Text). Proteomic analyses further confirmed higher levels of STM3175, YgiW, and QseB/C in the mutant T12 compared to WT cells (Fig H and Table N in S1 Text).

Closer inspection of its amino acid sequence revealed that STM3175 consists of 2 domains: an N-terminal AraC-like DNA-binding domain (DBD) and a C-terminal GyrI-like ligand-binding domain (LBD). In overexpression experiments, the LBD but not the DBD of STM3175 afforded resistance (Fig 1D and Fig F(C-D) and F(F) in S1 Text). Interestingly, the full-length protein appeared to confer higher resistance than the LBD alone. Expression of the LBD in an STM3175 mutant strain showed the same resistance level as when expressed in WT cells with intact STM3175 (Table E in S1 Text). In agar diffusion assays, both the full-length protein and the LBD alone neutralized the effects of albicidin (Fig I in S1 Text). When a 2-fold excess of albicidin was added to the STM3175 or LBD proteins before spotting, bacterial growth was clearly reduced, albeit less severely than in the control sample without protein (Fig I(C) and I(E) in S1 Text), excluding enzymatic modification or degradation of albicidin as mechanism of action for STM3175.

### STM3175 structure

The dual domain structure of a helix-turn-helix DNA-binding element combined with an effector binding domain is reminiscent of AlbA [20] (Fig J(A) in S1 Text), the MerR-like transcription factor that confers albicidin resistance in *Klebsiella oxytoca*. However, the affinity for albicidin and the dual domain structure appeared to be the only similarities. Not only is the sequence identity low (15%; Fig J(B) in S1 Text) but also the predicted secondary structure of the LBDs is markedly different. AlbA exclusively consists of helical elements, whereas STM3175 is composed of α-helices and β-sheets (Fig K in S1 Text). Circular dichroism (CD) spectroscopy of recombinantly expressed proteins confirmed the mixed content of α-helix and β-sheet in STM3175 with clearly reduced helical contributions when the DBD is not present (Fig L(A-C) in S1 Text).

These results were in excellent agreement with our crystal structure of full-length STM3175 (PDB-ID: 7R3W) where the N-terminal domain consists of 7 helices that form 2 helix-turn-helix DNA-binding motifs typically found in AraC DBDs [24] (Fig 1E and Fig M in S1 Text). The DBD is connected to the C-terminal LBD via a short helical element and a short linker (approximately 10 aa), which was not resolved in the electron density. The LBD shows the characteristic GyrI-like fold of 2 SH2 motifs with a pseudo 2-fold symmetry forming a central groove that is clearly visible in the crystal structure despite the relatively low resolution of 3.6 Å (Fig 1E). The dimensions of the groove (length approximately 30 Å, width approximately 10 Å, height approximately 12 Å) provide an ideal environment for accommodating compounds of linear architecture [25]. Despite presenting as a monomer in analytical gel filtration (Fig N in S1 Text), STM3175 forms domain-swapped dimers in the crystal, where the DBD of one polypeptide forms contacts with the LBD of a second molecule (Fig O(A-C) in S1 Text). The relative orientation of the N-and C-terminal domains of the 2 molecules is not identical, resulting in an asymmetrical dimer (Figs O(D) and O(E) in S1 Text).

When generating homology models by RoseTTAFold [26] and Phyre2 [27], the *E*. *coli* transcription factor Rob (PDB-ID: 1d5y) [28] and GyrI (PDB-ID: 1jyha) [25] were identified as highest-ranking structural homologs (100% and 99.9% confidence, respectively; Fig P in S1 Text). While the structures of the N- and C-terminal domains in the crystal structure and homology model were very similar (RMSD <1.5 Å), the relative domain orientation differed significantly. The homology models showed a more compact structure, similar to that of Rob bound to DNA (Fig P(A) in S1 Text). This flexibility in the relative domain orientation is consistent with STM3175 structure models by AlphaFold [29,30] (model available in AlphaFold Protein Structure Database under UniProt ID: Q8ZM00), where the predicted aligned error plot also suggests lower confidence in interdomain accuracy of the prediction. Rob and GyrI, as well as a number of other GyrI-like domain containing proteins, are also found when submitting the STM3175 crystal structure to the DALI server [31] to identify structurally similar proteins in the PDB. However, in contrast to other GyrI homologs, STM3175 lacks the highly conserved Glu residue in the center of the binding groove, and the charges in and around the groove are not exclusively negative as in most GyrI-like domains [32]. In STM3175, the groove is mostly lined with hydrophobic residues and extends into a tunnel toward the C-terminus of the LBD (Fig M in S1 Text).

### STM3175 albicidin binding

**Fig 2:**
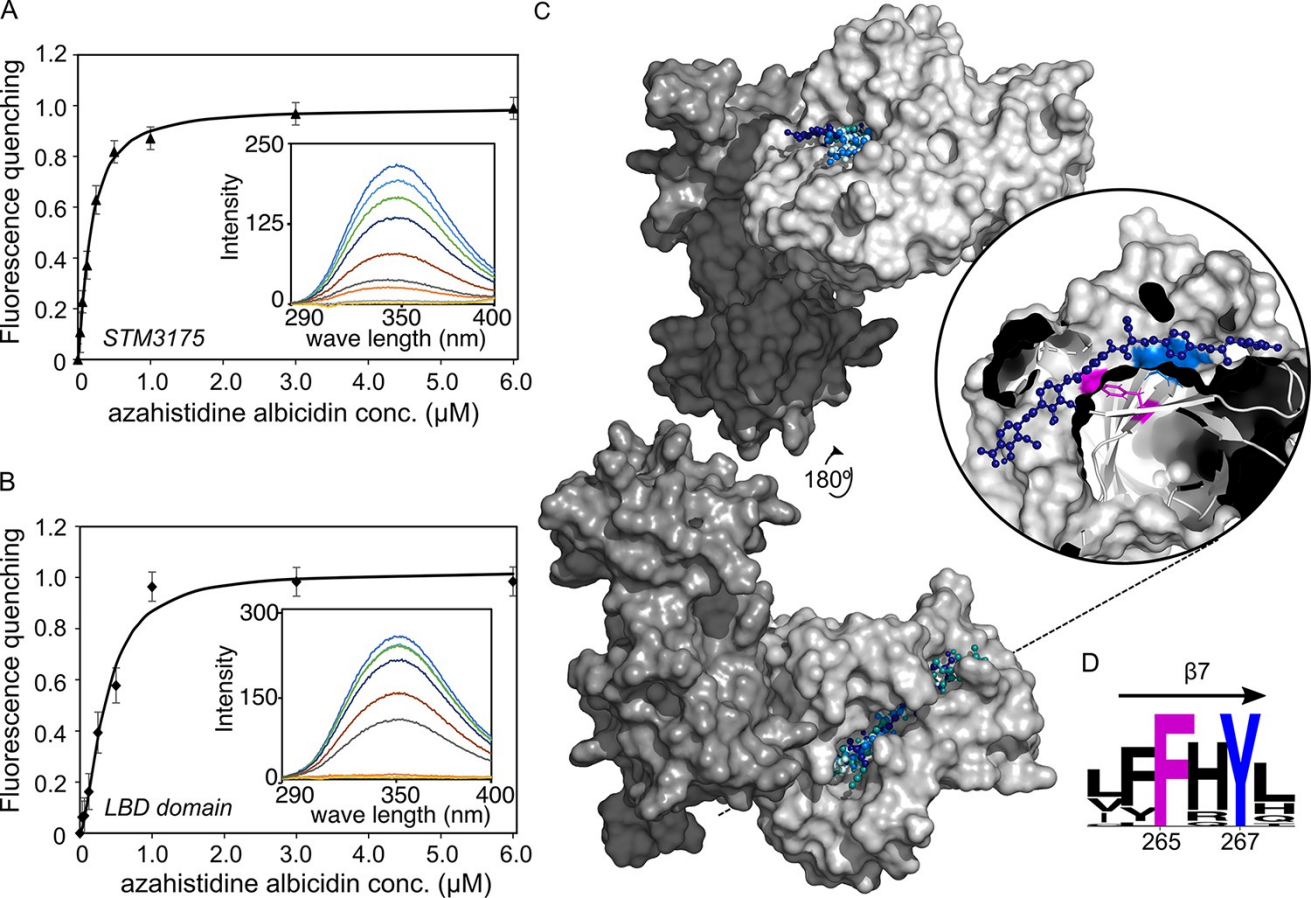
Albicidin binding to STM3175. The binding affinity of STM3175 and its LBD were determined by monitoring quenching of tryptophan fluorescence emission of STM3175 after addition of azahistidine albicidin. (**A**) Fluorescence quenching of STM3175 plotted as a function of increasing azahistidine albicidin concentration. Fitting of the decreasing fluorescence emission yielded a K_d_ of 0.17 ± 0.01 μM. (**B**) Fluorescence quenching of STM3175-LBD plotted against increasing azahistidine albicidin concentrations. Fitting of the data yielded a K_d_ of 0.35 ± 0.05 μM. Insets show emission spectra of STM3175 or the LBD with increasing azahistidine albicidin concentrations. (**C**) Surface model of the crystal structure of STM3175. DBD and LBD are colored in dark and light gray, respectively. The albicidin orientations of the top 4 best scoring models obtained with AutoDock Vina are shown in hues of blue. The inset shows cross-section through the binding pocket of the highest-ranking complex model with the conserved Phe265 highlighted in pink. The black dashed line indicates the cross-section plane. (**D**) WebLogo [33] depiction of amino acids in β-strand 7 using alignments of STM3175 homologs. The conserved Phe 265 and Tyr 267 are highlighted in pink and blue, respectively (see also Fig U in S1 Text). Source data are provided as a source data file (**S1 Data**). DBD, DNA-binding domain; LBD, ligand-binding domain.

Since several Trp residues are located in and around the binding groove, we monitored the interaction with albicidin by tryptophan fluorescence quenching experiments. To obtain affinity constants, albicidin was titrated to STM3175 or STM3175-LBD, and the concentration-dependent decrease in the fluorescence signal was measured. Fitting of the quenching data suggests that STM3175 binds albicidin with sub-micromolar affinity (K_d_ = 0.17 ± 0.01 μM; Fig 2A). The LBD alone binds with similar but somewhat lower affinity (K_d_ = 0.35 ± 0.05 μM; Fig 2B), suggesting that the DBD is not or only little involved in albicidin binding.

Attempts to crystallize the albicidin-STM3175 complex were unsuccessful, but molecular docking of albicidin to the crystal structure of STM3175 suggests a binding mode where albicidin is positioned in the central groove of the LBD (Fig 2C). In the best-ranked models of the complex, the N-terminal half of albicidin occupies the binding domain groove, which is a well-described binding site for other ligands of GyrI-like proteins [32]. The C-terminal building blocks D, E, and F extend through the tunnel toward the C-terminus of the LBD (Fig 2C). Curiously, in the domain-swapped dimers found in the crystal, the exit of this tunnel is blocked by one of the helices of the DBD (Fig M(C) in S1 Text). However, considering the low resolution of the X-ray structure, the exact positioning of albicidin in the binding groove cannot be determined with certainty by docking studies, and in some of the lower-ranked models, we also found albicidin in the opposite orientation (Fig Q(A) in S1 Text).

### STM3175 homologs in *E*. *coli*, *Vibrio*, and *Pseudomonas*

A BLAST search revealed homologs in various members of the Enterobacteriaceae family, including *Klebsiella*, *Klyuvera*, and *Citrobacter*. These homologs display the same transcription regulator di-domain structure with sequence identity ranging between 60% and 90%. The genomic context corresponds to that of *STM3175* in *Salmonella* with topoisomerase IV subunit A, QseB, and QseC located in the direct vicinity (Fig R(A-B) in S1 Text). Moreover, variants of the protein lacking the DBD, similarly to GyrI, are present in other Enterobacteriaceae genera such as *Escherichia* and *Shigella*. The *E*. *coli* homolog is known as YgiV (approximately 50% sequence identity, similar genomic context), and a search through NCBI records revealed a number (approximately 1,500) of database entries of proteins with the same name. In the present results are the short variant (160 aa) and the di-domain protein (288 aa) with the majority of hits in *E*. *coli* (short variant) followed by *Salmonella enterica* and *Klebsiella pneumonia* strains (long variants).

To investigate whether these homologs confer albicidin resistance and whether they are regulated by a similar GDA-based resistance mechanism, we conducted evolution experiments in *E*. *coli*. When exposed to increasing concentrations of albicidin, 11 out of 20 strains adapted to at least 8 μg mL^−1^ within 10 passages (Fig 1A). Nine strains harbored GDAs, again varying in length and copy number (Fig S(A) and Table B in S1 Text). An approximately 644-bp-long region was present in all GDAs, and, satisfyingly, it contained the *E*. *coli ygiV* gene (Fig S(B) in S1 Text). When the *E*. *coli ygiV* gene was expressed under control of an arabinose-inducible promoter in *S*. *Typhimurium*, we also observed elevated albicidin tolerance comparable to that conferred by the LBD of STM3175 alone (Fig T(A-B) in S1 Text).

AlphaFold and Rosetta both suggest that *E*. *coli* YgiV has a fold identical to that of the LBD of STM3175 with a binding groove sandwiched between 2 helices (AlphaFold model available in AlphaFold Protein Structure Database under UniProt ID: Q46866). Notably, the conserved Glu residue that is located at the base of the binding groove in most GyrI-like domains is also not present. In docking studies with the homology model, we obtained similar results as for STM3175 (Fig Q(B) in S1 Text) but with a preferred orientation of the albicidin carboxylic acid toward the C-terminus of helix1 (in STM3175, this would be the end of the groove opposite of the DBD).

We then sought to identify homologs from more distant bacterial relatives. A BLAST search using only the LBD of STM3175 identified single- and di-domain homologs in other Gammaproteobacteria, including *Vibrio* sp. and *Pseudomonas* sp. Because of their pathogenic potential, we decided to investigate whether the homologs in *Vibrio vulnificus* (YgiV-Vv; 44% identity to STM3175) and *Pseudomonas aeruginosa* (AraC-Pa-like; 48% identity) also conferred albicidin resistance. When the 2 di-domain homologs were expressed under control of an arabinose-inducible promoter in *S*. *Typhimurium*, we again observed elevated MICs, similar to those conferred by STM3175 (Fig T(C-D) in S1 Text).

Based on these results, it is likely that single- and di-domain STM3175 homologs in various gram-negative species can increase albicidin tolerance if present in sufficient copy numbers. Most homologs (>90% of 250 BLAST search results), including YgiV-Vv and AraC-Pa, have a Phe in place of the Glu residue usually found on β-strand 7 that forms the base of the binding groove (Fig U in S1 Text). Together with a similarly conserved Tyr, this Phe residue (Phe265 and Tyr267 in STM3175) forms an ideal interaction motif for the aminobenzoic acid blocks of albicidin (Fig 2D). MIC assays with STM3175 variants where the 2 aromatic residues at the base of the binding groove, F265 and Y267, were mutated to alanines, confirmed the critical role of these 2 amino acids at the binding site. The albicidin tolerance was drastically reduced in *S*. *Typhimurium* when STM3175 variants F265E, F265A, or the double mutant F265A/Y267A were expressed instead of the WT protein (Fig V in S1 Text).

### Specificity of the resistance mechanism

To evaluate the specificity of this resistance mechanism, the 6 evolved *S*. *Typhimurium* strains (S41, T01, T04, T05, T10, and T12) were challenged with compounds from different antibiotic classes, such as fluoroquinolones, tetracyclines, β-lactams, or sulphonamides. With MICs comparable to those of the WT strain, none of the evolved strains showed resistance against any of the tested antibiotics (Tables F and G in S1 Text). Interestingly, although albicidin and fluoroquinolones both target DNA gyrase [34], cross resistance was not observed in either our evolved strains or fluoroquinolone-resistant (FQR) *S*. *Typhimurium* strains [35] that were tested against albicidin (Fig W in S1 Text).

SbmC (GyrI) from *E*. *coli*, to which the LBD of STM3175 shows homology, protects cells from the gyrase poison microcin B17 [36]. However, overexpression of the SbmC homolog in *Salmonella* (71% sequence identity to the *E*. *coli* variant) showed only a minimal increase in albicidin tolerance (Fig X in S1 Text). Likewise, despite its homology to SbmC, evolved *E*. *coli* strains harboring GDAs of YgiV did not confer resistance against microcin B17 (Fig Y and Table H). Taken together, these results indicate that the STM3175-based resistance mechanism against albicidin is highly specific.

## Discussion

In our evolution experiments, we observed the emergence of GDAs that allow *S*. *Typhimurium* and *E*. *coli* to tolerate albicidin concentrations significantly exceeding those conferred by mutations in the well-studied nucleoside transporter Tsx. This high-level resistance resulted from the presence of several copies of the transcription regulator STM3175 in the genomes of evolved strains. GDAs allow rapid transcription regulation independent of transcription factors, which enables bacteria to quickly adapt to growth limiting factors [37] such as heat [38], lack of nutrients [39], or heavy metals [40].

Compared to point mutations, spontaneous duplications occur without selective pressure and at significantly higher rates (10^−4^–10^−2^/cell/division) with further increases of the copy number at rates of 10^−2^/cell/division [3,41]. This intrinsic genetic instability allows amplification-mediated gene expression tuning and enables populations to quickly respond to changes in environmental conditions [37,41]. The presence of GDAs can complicate treatment of bacterial infections as it can result in heteroresistance [42], in which subpopulations are less susceptible to the treatment, ultimately leading to treatment failure.

A number of different mechanisms are now known that can increase gene copy number in bacterial chromosomes. In principle, 2 main mechanisms are described: an amplification dependent or independent on the influence of the recombinase protein RecA [4]. We tested RecA dependence of STM3175 GDA formation by using a tsx/recA-deficient double mutant strain. Similar to the corresponding *tsx*-deficient parental strain, a significant increase in MIC upon evolution against increasing albicidin concentrations was detected. However, qPCR studies using *STM3175*-specific primers showed very clearly that the increase in MIC against albicidin was not accompanied by an increase in gene copy number of *STM3175* (Table I in S1 Text). Thus, albicidin-induced establishment of *STM3175* GDAs clearly follows RecA-dependent mechanisms. RecA-dependent mechanisms of GDA formation usually indicate the presence of repetitive sequences at the ends of duplicated regions. However, we did not detect any such sequences using extensive in silico analyses. It is known that initiation of GDA events sometimes proceeds from RecA-independent duplications These duplications are then used as repetitive sequences for subsequent RecA-dependent gene multiplications [3,43,44]. Future experiments are required to show whether this mechanism also plays a role in the formation of *STM3175* GDA events.

Frequently, the duplications clearly serve to increase the cellular levels of a gene product that can directly counteract the induced stress, such as efflux pumps in case of antibiotics or heavy metals [40]. In our arabinose-inducible expression systems, the albicidin-neutralizing effect of STM3175 is clearly dosage dependent. Our adapted *Salmonella* and *E*. *coli* strains gain up to 15 copies of the gene and can tolerate more than a 100-fold higher albicidin concentrations However, GDAs are intrinsically unstable, and, in the absence of selective pressure, they are generally rapidly resolved to ameliorate the inherent fitness cost [3,45]. Investigations on the kinetics of *STM3175* GDA reversal are a particularly interesting aspect. Our studies and those of others show that this is a highly individual process that occurs primarily at the single-cell level [46]. Advances in innovative sequencing technologies, such as MinION nanopore sequencing, will allow us in the future to delve deeper into the mechanisms of formation and reversion of GDAs. Ultimately, these insights will allow us not only to describe in detail kinetics of bacterial subpopulations but also to predict the emergence of successful (antibiotic resistance) clones.

Under ongoing selective pressure, GDAs can provide a basis for the evolution of genes with new functions, as the additional gene copies could potentially evolve independently, acquiring new functions or specificities [40,47], e.g., to diversify or specialize an existing resistance mechanism or generation of new fusion proteins. In this study, it is conceivable that overexpression of STM3175 by GDA events paves the way for fixed, genetic albicidin resistance by subsequent mutations in the Tsx nucleoside channel, as shown for *E*. *coli* [22].

It appears unlikely that YgiV and STM3175 are resistance traits that evolved specifically to counteract albicidin. The lack of cross-resistance in our tests with numerous antibiotics and the high affinity for albicidin, however, suggest specificity of the mechanism. In this regard, it would be key to see how the ex vivo-determined albicidin binding affinities compare to intracellular albicidin levels. However, due to various challenges, e.g., hydrophobicity, we were not able yet to determine these with confidence. In the MIC assays, albicidin concentrations range from 0.0012 μM to 12 μM, which is in a similar order of magnitude as the dissociation constants we obtained from Trp fluorescence quenching experiments (0.17 μM for STM3175 and 0.35 μM for the LBD). However, we doubt that the STM3175 binding is sufficient to trap all albicidin molecules sufficiently in order to prevent inhibition of gyrase, rather that other additional mechanisms are involved, e.g., the transcriptional up-regulation of efflux systems or STM3175-mediated autoregulation. On the other hand, GyrI-like domain containing transcription factors like STM3175 have been implicated in polyspecific binding and multidrug resistance [40,47], and STM3175 might have the ability to protect cells from other stressor molecules. A role in DNA protection has been described for the homolog GyrI. Interestingly, the protein GyrI, which was originally discovered as a gyrase inhibitor, protects DNA gyrase from the peptide toxins microcin B17 and CcdB and offers partial protection from quinolones [32]. GyrI also imparts resistance against alkylating agents mitomycin C and N-methyl-N-nitro-N-nitrosoguanidine, which act independently of gyrase [48].

DNA gyrase inhibitors generally fall into 2 categories: those that inhibit the binding of ATP and interrupt supercoiling (e.g., aminocoumarins or cyclothialidines) and those that trap enzyme–DNA intermediates (e.g., quinolones, CcdB, and microcin B17). Albicidin belongs to the second category [3–5]; hence, a resistance mechanism comparable to that of microcin B17 seems reasonable. However, the molecular details behind the mechanism of action of albicidin have not yet been determined, and this similarity to microcin B17 and CcdB might provide further insights.

Members of the GyrI superfamily are prevalent among bacteria, archaea, and eukaryotes [17]. PFAM lists GyrI-like family members as stand-alone domains or fused to other functional domains, such as DNA-binding or enzymatic domains. In our experiments, we observed higher resistance in overexpression experiments of full-length STM3175 compared to YgiV or STM3175 without DBD. Notably, this difference was not observed in agar diffusion experiments, which allows 2 possible explanations: first, full-length STM3175 can bind DNA and activate other defense mechanisms, such as an elevated expression of efflux pumps (as shown for the closely related transcription factor Rob) [49], or second, STM3175 can undergo autoregulation leading to a potentiation of the protein itself. It would be interesting to see in future experiments which promoter regions are recognized by the DBD and which genes are directly affected.

A number of structures of proteins with GyrI-like domains are available in the PDB, but only a few of the deposited structures are of di-domain proteins: ROB [28], BmrR and EcmrR, 2 multidrug sensing regulators of the MerR family that consist of an N-terminal helix-turn-helix DBD with a dimerization motif and a GyrI-type LBD. The domain architecture of STM3175 resembles that of ROB, which also consists of an N-terminal AraC DBD fused to a GyrI-like domain [28]. Despite its relatively low resolution, the crystal structure of STM3175 agreed very well with predictions and available GyrI-family structures in the PDB. And while we were not able to obtain crystals of albicidin bound to STM3175, our models suggest a binding mode that is consistent with structural data of various drug-like compounds in complex with GyrI-like proteins [32].

In summary, we demonstrated that exposure of *S*. *Typhimurium* and *E*. *coli* to increasing concentrations of the gyrase poison albicidin results in rapid adaption via chromosomal duplication–amplification events. The affected region harbors the GyrI-like domain containing transcription regulator STM3175 (YgiV), which we identified as a critical factor involved in high-level resistance against albicidin. The protein binds albicidin with high affinity in an equimolar stoichiometry. We further showed that this resistance mechanism/gyrase protection mechanism is ubiquitous in Enterobacteriacea with STM3175 homologs conferring resistance in *Escherichia*, *Vibrio*, and *Pseudomonas*.

## Supporting information

S1 Text **Fig A. Agar diffusion assays using natural albicidin.** (**A**) Assay scheme illustrating the sample arrangement on agar plates. The negative and positive controls contain only protein (−) or albicidin (+) in buffer with 5% DMSO, respectively. (**B**) STM3175 with albicidin in a 1:1 molar ratio (in triplicates I-III). **Fig B. Reduction in GDA copy number after albicidin removal.** (**A**) Scheme of the experimental setup. The respective evolved strains harboring multiple GDAs were incubated in LB medium without albicidin supplementation. Bacteria were diluted in fresh growth medium every 24 h. After 6 days, bacteria were harvested and a qPCR using STM3175-specific primers were performed to determine the STM3175 copy number. (**B**) The fold change value represents the relative ratio of the GDAs of the evolved or back-evolved strain (3 different clones) to the unevolved parent strain. Normalization done using the housekeeping gene *trp*. Fig (A) was created with BioRender. Source data are provided as a source data file (**S1 Data**). **Fig C. MIC detection of evolved *S*. *Typhimurium* strains.** Adaptation to albicidin in 14/90 strains after overnight incubation with the 4-fold albicidin wild type MIC (WT; ATCC 14028; black bar). Nine evolved strains (dark gray bars) showed mutations in the *tsx* gene and a comparable MIC to the *tsx* mutant (white bar, 0.25 μg mL^−1^). Five evolved strains showed 100% *tsx* gene identity to the WT. The strain S41 (red bar) has an increased MIC of 2 μg mL^−1^, corresponding to an increase by almost 70-fold to the WT (0.00156 μg mL^−1^). This strain was investigated in further experiments. Data represent mean and standard deviations of 6 biological replicates that were performed in 3 technical replicates (WT, Δ*tsx*, S41). The data of the other strains represent means and standard deviations of 2 biological replicates that were performed in 3 technical replicates. Strains without error bar have the same result in every single experiment. Source data are provided as a source data file (**S1 Data**). **Fig D. Albicidin high-level resistance resulting from GDAs in evolved *S*. *Typhimurium*.** (**A**) Evolution of GDAs in 6 strains after treatment with albicidin (S41: 0.06 μg mL^−1^ albicidin for 24 h, light gray bar; T-strains: increasing albicidin conc. From 0.125 to 20 μg mL^−1^ in 9 passages, dark gray bars) results in multiple copy regions of varying length. The same approximately 2,200 bp segment is present in each copy region in all evolved strains (cyan). T12 with 7 copies of a region is shown as example. (**B**) The amplified region in T12 contains the genes for topoisomerase IV subunits A and B (*topoIV A*, *topoIV B*), QseBC, STM3175, YgiW, YgiN, modulator of drug activity B (*mdaB*), and 2 ORFs for hypothetical proteins. **Fig E. MIC determination of knockout mutants.** (**A**) *S*. *Typhimurium* knockout mutants of *qseBC* (dark gray) and *STM3175-ygiW* (light gray) show the same azahistidine albicidin tolerance as their respective parent strain: WT (black) or Δ*tsx* mutant (white). The data represent means and standard deviations of 3–5 biological replicates that were performed in 3 technical replicates. Strains without error bar have the same result in every single experiment. (**B**) For a selection of mutant strains, CFU counts were determined in parallel to MIC measurements by optical density. The data represent means and standard deviations of 2 biological replicates that were performed in 3 technical replicates. Source data are provided as a source data file (**S1 Data**). **Fig F. MIC curves and CFU counts of the STM3175 operon and domains.** (**A**, **B**) Comparison of noninduced and arabinose-induced expression systems of genes in the GDA region shows increased azahistidine albicidin tolerance when expression of STM3175 is induced. (**C**, **D**) After supplement of 5 mM arabinose, the MIC was increased for full-length STM3175 and, to a lesser extent, its LBD. (**E**, **F**) CFU counts according to MIC of arabinose induced expression systems of genes in the GDA region and STM3175-domains (E vs. B; F vs. D). The data represent means and standard deviations of 3 biological replicates that were performed in three technical replicates. Source data are provided as a source data file (**S1 Data**). **Fig G. MIC determination for *S*. *Typhimurium* with STM3175 overexpression.** Overexpression of STM3175 was induced by increasing arabinose concentrations. The data represent means and standard deviations of 4 biological replicates that were performed in 3 technical replicates. Source data are provided as a source data file (**S1 Data**). **Fig H. Up-regulation of the GDA region.** RNA sequencing and proteomics data of evolved *S*. *Typhimurium* strain T12 show elevated mRNA and protein expression levels for genes in the GDA, particularly STM3175 and YgiW. Proteins indicated by a cross were not identified in the proteomics analysis. **Fig I. Agar diffusion assays.** STM3175 and LBD (GyrI-like domain) with azahistidine albicidin. (**A**) Assay scheme illustrating the sample arrangement on agar plates. The negative and positive controls contain only protein (−) or azahistidine albicidin (+) in buffer with 5% DMSO, respectively. (**B**-**E**) STM3175 and LBD with albicidin in a 1:1 or 1:2 molar ratio (in triplicates I-III). **Fig J. Domain structure and sequences of STM3175 and AlbA.** (**A**) Domains identified by the NCBI conserved domains search tool [2]⁠ are highlighted in bold. (**B**) CLUSTAL Omega sequence alignment of STM3175 and AlbA. **Fig K. Secondary structures of STM3175 and AlbA.** Secondary structures predicted from the amino acid sequences by the PSIPRED Server. α-helices are colored in yellow; β-sheets are shown in blue. Amino acid residues in the ligand-binding domains are underlined. **Fig L. Circular dichroism spectroscopy of STM3175.** (**A**) SDS-PAGE with His_6_-STM3175 (FL) and His_6_-STM3175-LBD (LBD) after Ni-NTA purification. (**B**) CD spectrum of His_6_-STM3175 in Tris buffer (black) and with K2D reconstructed spectrum (gray). (**C**) CD spectrum of the ligand binding domain in Tris buffer. MRE = mean residue ellipticity. Source data are provided as a source data file (**S1 Data**). Original scans for Fig (A) can be found as supporting information (**S1 Fig**). **Fig M. STM3175 surface analysis.** (**A**) STM3175 crystal structure with (**B**) hydrophobic residues colored in orange and polar amino acids shown in light blue and (**C**) negatively charged amino acids in red and positively charges residues in blue. **Fig N. Analytical size exclusion chromatography.** (**A**) Chromatogram of an analytical size exclusion gel filtration run of STM3175 on a Superdex S200 increase 3.2/300. STM3175 (with a concentration of 30 μM in 50 μl in gel filtration buffer) was analyzed with a flow rate of 0.04 ml/min. (**B**) Calibration curve of the column with thyroglobulin (670 kDa), γ-globulin (158 kDa), ovoalbumin (44 kDa), and myoglobin (17 kDa) shown as blue circles. The apparent molecular weight of STM3175 was calculated from the elution volume using the linear regression (blue line). The apparent molecular weight of STM3175 is 43.5 kDa (data point shown in red). **Fig O. Spatial organization of STM3175 in the crystal structure.** (**A**) Arrangement of 2 domain-swapped dimers in the crystal. (**B**) Schematic of the monomer organization in a unit cell with hexagons symbolizing the ligand-binding domains and ellipses as HTH domains. (**C**) A helix (highlighted in orange) of the DBD of monomer A occupies the C-terminal end of the binding groove on the LBD of monomer C. (**D**) Alignments of the LBD of the 4 monomers show 2 distinct domain orientations. Monomers A and D resemble each other and monomers B and C align. (**E**) Each dimer consists of 2 monomers with different conformations, but alignments show that the 2 dimers are not identical. Source data are provided as a source data file (**S1 Data**). **Fig P. STM3175 homology model and modeling templates.** (**A**) Crystal structure of the highest scoring template, transcription factor ROB, bound to its cognate DNA in a tertiary complex (PDB-ID: 1d5y). (**B**) Crystal structure of the highest scoring template for the ligand-binding domain SbmC (GyrI; PDB-ID: 1jyh). (**C**) Robetta homology model of STM3175. The DBDs are colored in gray, LBD in cyan. In STM3175, SH2 subunits of the pseudo-dimeric motif are colored in pale cyan and green. **Fig Q. LBDs of STM3175 and *E*. *coli* YgiV with albicidin.** (**A**) Conformation of albicidin in the 4 best-ranked models in AutoDock Vina with the crystal structure of STM3175. The top-ranked conformation is shown in dark blue. (**B**) Rosetta model of *E*. *coli* YgiV docked with albicidin using HADDOCK. The 4 best-ranked models are shown with the top-ranking albicidin conformation shown in dark red. **Fig R. Enterobacteriaceae family tree and genomic context of topoisomerase IV subunits.** (**A**) Genera with mono- or di-domain STM3175 homologs in the same genomic context are highlighted in green. Genera where YgiW was present but no STM3175 homolog are indicated with an asterisk. The phylogenetic tree was adapted from Hata and colleagues [10]⁠. (**B**) Genomic context of the 2 subunits of topoisomerase I, *topoIV A* and *topoIV B*. *ygiW* is shown in gray. *LysR* = LysR family transcriptional regulator; *MO* = antibiotic biosynthesis monooxygenase; *O red* = NAD(P)H-dependent oxidoreductase. Genomic sequences were obtained from the NCBI database with the following accession numbers: *E*. *cloacae*: NZ_CP009756.1, *C*. *youngae*: NZ_GG730303.1, *Y*. *rohdei*: NZ_CP009787, *S*. *plymuthica A9*: NC_015567.1, *C*. *dublensis*: NZ_CP012266.1, *E*. *persicina*: NZ_CP082141.1. **Fig S. Gene duplication and amplification (GDA) in *E*. *coli* strains with albicidin hyperresistance.** (**A**) Mapping of GDA strains after treatment with albicidin (increasing albicidin conc. from 0.0156 μg mL^−1^ to 8 μg mL^−1^ in 10 passages), which are different in copy number and size (Table B). (**B**) The common approximately 600-bp-long region (blue) includes the gene *ygiV*. **Fig T. Comparison of noninduced and arabinose-induced expression systems of STM3175 homologs from *E*. *coli*, *V*. *vulnificus*, and *P*. *aeruginosa* in *S*. *Typhimurium*.** (**A**, **B**) Arabinose induction results in elevated albicidin MIC in *S*. *Typhimurium* cells where the homolog YgiV-Ec from *E*. *coli* is expressed but not when YgiW-Ec is expressed. (**C**, **D**) Arabinose-induced overexpression of the STM3175 homologs YgiV-Vv from *V*. *vulnificus*, its LBD (YgiV-LBD-Vv), and AraC-Pa from *P*. *aeruginosa* leads to increased albicidin MICs. The increased MIC for YgiV-Vv even without arabinose induction is due to its DBD, which is an AraC homolog and might results in autoregulation under albicidin treatment. Sensitivity of Ygiv-Vv to albicidin without arabinose induction is restored after cloning of only LBD of YgiV. The data represent means and standard deviations of 3 biological replicates that were performed in 3 technical replicates, except YgiW-Ec, which was performed in 2 biological and 3 technical replicates. Source data are provided as a source data file (**S1 Data**). **Fig U. Alignments of STM3175 homologs.** Shown are representatives from different genera of the top 100 homologs identified by BLAST of the LBD against the RefSeq database excluding *Salmonella* sp. Proteins investigated in this study are indicated in red. Amino acids with 100% consensus are shown in red, and the beta-strand forming the base of the binding groove is highlighted by a red box. **Fig V. Influence of point mutations in the putative albicidin binding pocket of the STM3175-LBD on albicidin resistance.** (**A**) Crystal structure of STM3175 with its 2 domains. The residues in the binding pocket of the LBD that were mutated are highlighted in pink. (**B**) MIC assay results of the arabinose-inducible vector pBAD30 (control strain), STM3175 overexpression strain, and introduced mutations (*F265E, *W244A, *F265A, *Y267A, *F265A-Y267A) in the binding pocket of STM3175 overexpression strain. **Fig W. MICs of albicidin and ciprofloxacin.** Determination of the MIC for the wild type (WT, ATCC 14028), the albicidin evolved strain (S41), and 4 high fluoroquinolones (FQ)-resistant strains (FQR1–4) for (**A**) albicidin and (**B**) ciprofloxacin in 96-well plates. The error bars represent mean and standard deviations of 3 biological replicates that were performed in 3 technical replicates (except FQR1–4 for ciprofloxacin, which represent mean and standard deviations of 3 technical replicates). Strains without error bar have the same result in every single experiment. Source data are provided as a source data file (**S1 Data**). **Fig X. Comparison of arabinose-induced expression system of *S*. *Typhimurium* SbmC and empty vector in *S*. *Typhimurium*.** (**A**) MIC determination without arabinose and 5 mM arabinose in empty vector control pBAD30, (**B**) in pBAD30::*sbmC*. The error bars represent mean and standard deviations of 2 biological replicates that were performed in 3 technical replicates. Source data are provided as a source data file (**S1 Data**). **Fig Y. Agar diffusion assay with MccB17 and evolved *E*. *coli* and *S*. *Typhimurium* strains.** At concentrations of 10 mg/ml, no difference in susceptibility toward MccB17 was observed between WT or evolved *E*. *coli* strains E08 and E10. *S*. *Typhimurium* WT strain was not susceptible to MccB17 and neither was the evolved strain T12. **Table A. Genetic changes in albicidin resistant *Salmonella* isolates. Table B. Summary of evolved *S*. *Typhimurium* and *E*. *coli* strains harboring a GDA region. Table C. Genetic changes in albicidin-resistant *Δtsx Salmonella* isolates. Table D. Summary of evolved *S*. *Typhimurium* strain T12 after growth without antibiotic pressure. Table E. MIC values of STM3175-LBD of noninduced and induced arabinose expression in different strains. Table F. Antibiotic susceptibility testing of wild-type strain (WT) and the respective evolved strain (S41) for 24 different antibiotics.** Abbreviations: **R, resistant. Table G. Antibiotic susceptibility testing of Δ*tsx* mutant strain and the respective evolved strains for 24 different antibiotics.** Abbreviations: **R, resistant. Table H. Susceptibility testing against microcin B17 of *E*. *coli* and *S*. *Typhimurium* strains.** *contains *SbmC* gene. **Table I. RecA dependence in GDA formation.** Evolution of *recA*^*+*^ (T-strains) and *recA*^*—*^strains (TR-strains) to an albicidin concentration of 2.5 μg/ml. * The foldchange value represents the relative ratio of the GDAs of the evolved strains to the unevolved originals strain. The foldchange (ΔΔC_T_) results from the relative value (ΔC_T_) of the evolved strain minus the relative value of the unevolved original strain, which were normalized to the ΔC_T_−value of the housekeeping gene *trp* beforehand. **Table J. Strains and plasmids used in this work.** Abbreviations: Nal^R^, nalidixic acid resistance; Cb^R^, carbenecellin resistance; Kan^R^, kanamycin resistance; Ap^R^, ampicillin resistance; SGSC, Salmonella Genetic Stock Centre; DB, DNA-binding domain; LBD, ligand-binding domain; Ec, *Escherichia coli;* Vv, *Vibrio vulnificus*; Pa, *Pseudomonas aeruginosa*. **Table K. Primer with their sequence and target region used in this work.** *GAAAACCTGTATTTTCAGGGC = TEV site. Abbreviations: DB, DNA-binding domain; LBD, ligand-binding domain; MCS, multiple cloning site. **Table L. STM3175-constructs cloned in this work.** Abbreviations: DB, DNA binding domain; LBD, ligand-binding domain. STM3175 coding sequence: M_0_NDLISAAYSERLRRVCDHIERHLDEPLSIEALSRMAHSSPFHFHRQFTTWSGLPLYRYIQWLRLRRASWRLAFNPQDKVIDIALDAGFQNPESFTRAFKTAFGQSPRRFRQSP_114_DWLAWHQRVPKLALQEQHV_133_MDVKIVEFPPTRVAMLTHLGHPDKVNASAAKFIAWRRETGQSPIASSQTFGIAWHDPQTTPPAQFRFDICGSVRQPIAENDVGVVNSEIPGGRCAVVRHQGSLDSLPESVWYLFREWLPASGETPRDFPVFFQYLNFVHEVAEHELLTDIYLPLR_288._ **Table M. Diffraction data collection, refinement, and validation statistics.** ^a^ Data for the highest resolution shell in parenthesis. ^b^ R_meas_(I) = ∑_h_ [N/(N-1)]^1/2^ ∑_i_ │I_*i*h_—<I_h_>│ / ∑_h_∑_i_ I_*i*h_, in which <I_h_> is the mean intensity of symmetry-equivalent reflections h, I_*i*h_ is the intensity of a particular observation of h, and N is the number of redundant observations of reflection h. [50]. ^c^ CC_1/2_ = (<I^2^> − <I>^2^) / (<I^2^> − <I>^2^) + σ^2^_ε_, in which σ^2^_ε_ is the mean error within a half-dataset [51]. ^d^ RMSD–root mean square deviation. ^e^ calculated with PHENIX [52]. ^f^ calculated with MOLPROBITY [53]. ^g^ Clashscore is the number of serious steric overlaps (>0.4) per 1,000 atoms [53]. Table N. List of 2063 quantified proteins from *S*. *Typhimurium* detected in wild-type strain (*n =* 5) and evolved T12 strain (*n* = 4*). The mass spectrometry proteomics data have been deposited to the ProteomeXchange Consortium via the PRIDE partner repository with the dataset identifier PXD031944.
(DOCX)

S1 Data Source data for the main figures (Figs 1C, 1D, 2A and 2B) and supporting figures (Figs B, C, E(AB), F(ABCDEF), G, L(BC), T(ABCD), W(AB), and X(AB)).
(XLSX)

S1 Fig Raw image. (TIF)

## Abbreviations

AMR: antimicrobial resistance
CD: circular dichroism
CFU: colony-forming unit
DBD: DNA-binding domain
FQR: fluoroquinolone-resistant
GDA: gene duplication–amplification
LBD: ligand-binding domain
MIC: minimum inhibitory concentration
SNP: single nucleotide polymorphism
WT: wild-type
