# Supplementary figures and images for: Gene amplifications cause high-level resistance against albicidin in gram-negative bacteria
Source: PLoS Biol. 2023 Aug 10;21(8):e3002186. doi: 10.1371/journal.pbio.3002186 (PMC10414762; doi:10.1371/journal.pbio.3002186)

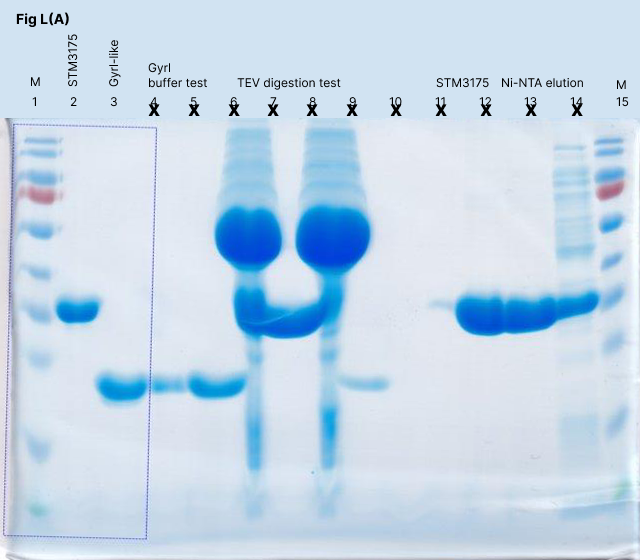

Supplement: S1 Fig — (TIF) [file pbio.3002186.s003.tif]
